# Mechanism of carbon nanotube growth in expanded graphite via catalytic pyrolysis reaction using carbores P as a carbon source

**DOI:** 10.3389/fchem.2023.1260099

**Published:** 2023-10-19

**Authors:** Yilong Wang, Wenli Zhang, Yuejun Chen, Xiongfeng Zeng, Jiankun Huang, Hengyong Wei, Junbo Tu

**Affiliations:** ^1^ College of Mining Engineering, College of Materials Science and Engineering, North China University of Science and Technology, Tangshan, China; ^2^ Tangshan Guoliang Special Refractory Limited Company, Postdoctoral Workstation, Tangshan, China

**Keywords:** carbon nanotubes, catalytic pyrolysis, DTA-TG-MS, growth mechanism, expanded graphite (EG)

## Abstract

Carbon nanotubes (CNTs) had potential applications in energy conversion and storage devices, and it could be prepared by expanded graphite loaded with catalyst at high temperature, however, the mechanism of carbon nanotube growth in expanded graphite need further confirmation. In this work, carbon nanotubes’ *in situ* growth in expanded graphite (EG) were prepared via catalytic pyrolysis reaction using carbores P as a carbon source and Co(NO_3_)_3_•6H_2_O as a catalyst. The results of X-ray diffraction (XRD), scanning electron microscope (SEM) and energy dispersive X-ray spectroscope (EDS) indicated the carbon nanotubes could generate in, EG with the presence of carbores P as a carbon source and cobalt nitrate as a catalyst. More interestingly, the growth mechanism of carbon nanotubes could be concluded by the results of differential thermal analysis-thermogravimetry-mass spectrometry (DTA-TG-MS) and X-ray photoelectron spectroscopy (XPS) analysis. The pyrolysis products of carbores P were mainly hydrocarbon gas such as CH_4_ gas, which reacts with Co(NO_3_)_3_·6H_2_O catalyst to reduces CoO_x_ to Co particles, then the carbon form pyrolysis was deposited the on the surface catalyst Co particles and, after continuous solid dissolution and precipitation, carbon nanotubes were at last generated in EG at last.

## 1 Introduction

Expanded graphite (EG) was first prepared in the early 1860s by mixing natural graphite with strong oxidants (Xing et al., 2022). EG possesses a loose and porous vermicular morphology, which leads to a large surface area. Hence, the expanded graphite is also named worm graphite. Expanded graphite as a kind of remarkable and new carbon material has excellent corrosion resistance, high temperature resistance, high thermal conductivity, a light weight, good adsorption, and insulation resistance. Therefore, it has been widely used in the fields of hydrogen storage, fuel cells, sensors, catalysts, adsorbents, medicine, and refractories (Yang et al., 2011; Li et al., 2013; Li et al., 2010; Qi et al., 2010).

Taking the application of, EG in refractories as an example, Shantanu K Behera blended Al_2_O_3_-C slide gate plate refractories using expanded graphite as the C source (Behera and Mishra, 2015). Apart from positive oxidation resistance, the refractories exhibited good hot and cold strength as well. In 2015, Wang researched the effect of the reactivity and porous structure of, EG on microstructures and the properties of Al_2_O_3_-C refractories (Wang et al., 2015). The results showed that the mechanical properties and oxidation resistance of the Al_2_O_3_-C refractories had been improved via the addition of, EG. Subsequently, Wang synthesized a boron- and nitrogen-doped expanded graphite as efficient reinforcement for Al_2_O_3_-C refractories (Wang et al., 2017). Analogously, they also studied Al_2_O_3_-C refractories using silicon hybridized expanded graphite as an addition to enhance their mechanical properties (Wang et al., 2018). In addition, the MgO-C refractories containing expanded graphite were investigated for their thermo-mechanical properties and oxidation resistance (Zhu et al., 2013; Subham Mahato and Behera, 2014; Mahato and Behera, 2016).

Some researches found that CNTs could be formed in refractory materials containing expanded graphite. Furthermore, the composites of CNTs and expanded graphite have the significance of synergistic toughening for refractory materials. Ming Luo et al. researched the Al_2_O_3_-C refractories containing CNTs, and found that the mechanical properties, such as the cold modulus of rupture and flexural modulus, were better than that of the materials without the CNTs (Luo et al., 2013). Similarly, Ming Luo et al. investigated carbon nanotubes *in situ* and ceramic whiskers in Al_2_O_3_-C refractories with the addition of Ni-catalyzed phenolic resin. The results also indicated that the formation of the CNTs and the ceramic whiskers lead to the enhancement of mechanical properties (Luo et al., 2012a). Additionally, Ming Luo et al. thought that the strength and toughness of refractory materials could be enhanced via introducing the CNTs/EG (Luo et al., 2012b).

Up to now, chemical vapor deposition has been widely used in the growth of carbon nanotubes in expanded graphite. Expanded graphite loaded with catalyst is heated at a high temperature, and the catalyst on its surface would react with hydrocarbon gases such as acetylene to form carbon nanotubes in expanded graphite. For example, Jianguo Zhao et al. prepared carbon nanotubes in the pores of expanded graphite via chemical vapor deposition using acetylene as the carbon source (Zhao et al., 2009). In addition, Baoyan Xing et al. successfully prepared CNTs in, EG through the *in situ* synthesis method by selecting naphthalene as the carbon source (Xing et al., 2022). Although the carbon source for carbon nanotubes growth in expanded graphite has generated great interest, the growth mechanism of carbon nanotubes has rarely been proven by experimental methods.

Carbores P is a carbonaceous powder with very high coking yield and softening point (>220°C), and it provides binder properties for resin-bonded magnesia-carbon (MgO-C) or dolomite bricks and alumina-carbon (Al_2_O_3_-C) refractories. And Carbores P was suited for use as a precursor of CNTs.

More interestingly, CNTs have a large surface area, enabling increased electrochemical accessibility and mechanical, chemical, and electrochemical stability, which creates the potential for CNTs to be used as a supplemental material for energy conversion and storage devices (Iqbal et al., 2019).

In this work, the cheap carbores P was used as a carbon source and Co(NO_3_)_2_•6H_2_O as a catalyst to form carbon nanotubes in expanded graphite through catalytic pyrolysis reaction, and argon was used as a protective atmosphere. The feasibility of the catalytic cleavage of carbores P to form carbon nanotubes was investigated, and the growth mechanism of the carbon nanotubes is discussed.

## 2 Materials and methods

### 2.1 Synthesis of CNTs in EG

**FIGURE 1:**
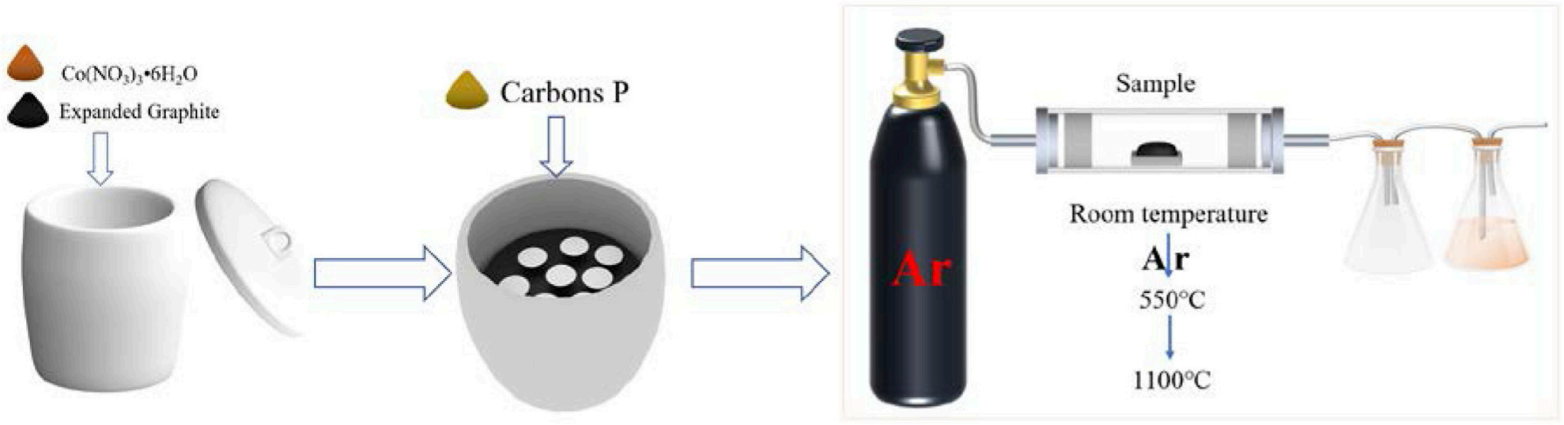
Scheme of the reactor setup.

Carbores P was taken as the carbon source. Co(NO_3_)_3_•6H_2_O was used as the catalyst that was 1.0 wt% of expanded graphite. Firstly, the expanded graphite and the catalyst Co(NO_3_)_3_ water solution were thoroughly mixed, and were placed in a corundum crucible post drying. Then, graphite paper was placed in the corundum crucible. After that, carbores P was put on the graphite paper and then heated to 550°C in a furnace using argon as a protective atmosphere. Next, the flowing argon was closed, and the sample continue to be heated at 1,100°C for 3 h at a rate of 3°C/min and then cooled down with the furnace. The scheme of the reactor setup is shown in Figure 1. The sample was classified into three types, as listed in Table 1.

**TABLE 1:** Dosage of raw material.

	Sample 1 (S1)	Sample 2 (S2)	Sample 3 (S3) (g)
Expanded graphite	0.06 g	0.06 g	0.06
Catalyst cobalt nitrate	0.0009 g	-	0.0009
Carbores P	-	15 g	15

### 2.2 Characterization

The phase composition of all samples was analyzed by D/MAX2500PC X-ray diffractometer (XRD). The scanning angle and scanning speed were 10–80° and 10°/min, respectively. The radiation source target of the diffractometer was the Cu-target Kα. The S-4800 field emission scanning electron microscope was equipped with an energy dispersive spectrometer (EDS) and was used to observe the morphology of the synthetic sample and the quantity of sample. Differential thermal analysis-thermogravimetry-mass spectrometry (DTA-TG-MS, NETZSCH STA 449F5, Germany) of the sample was carried out. The chemical state was tested by X-ray photoelectron spectroscopy (XPS, PHI5300C, United States).

## 3 Results and discussion

### 3.1 Phase analysis

**FIGURE 2:**
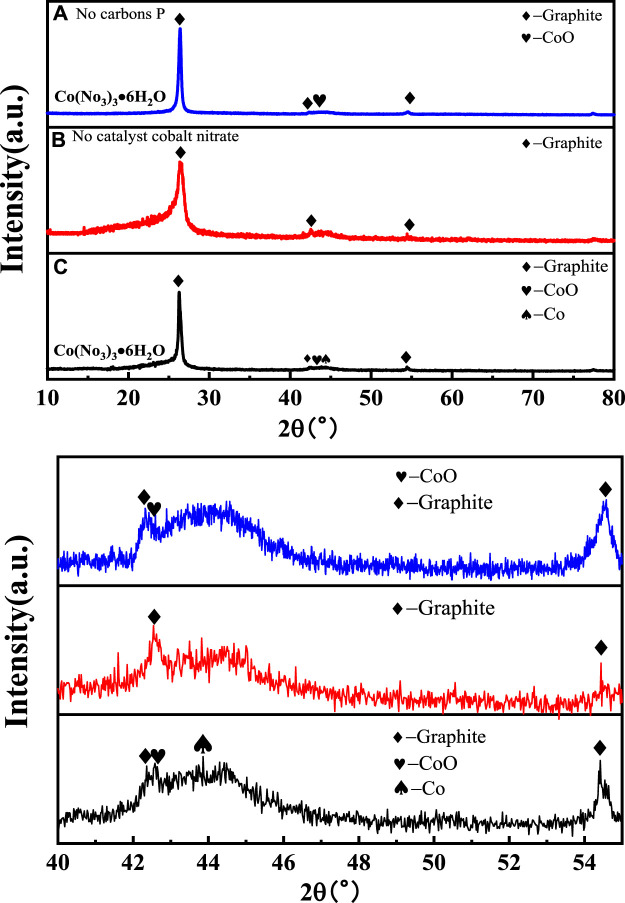
XRD spectra of sample 1 **(A)**, sample 2 **(B)**, and sample 3 **(C)**.

The phase composition of all samples was analyzed by XRD, and the results are presented in Figure 2. All samples showed a strong diffraction peak at 26.5°, which is a typical feature of the (002) crystal plane diffraction of graphitic material. There was a visible CoO phase in samples 1 and 3. Sample 3 had a sharper peak corresponding to the (200) plane of the CoO phase than that of sample 1. It could be obtained from the local magnification figure that there was a trace phase of the cobalt metal according with 44.2° in sample 3. It is reasonable that the phase differences of those samples should be related to the change of the Co element in the catalyst Co(NO_3_)_3_•6H_2_O during the catalytic process (Ince Yardimci et al., 2020; Saconsint et al. et al., 2022).

### 3.2 Microstructure analysis

**FIGURE 3:**
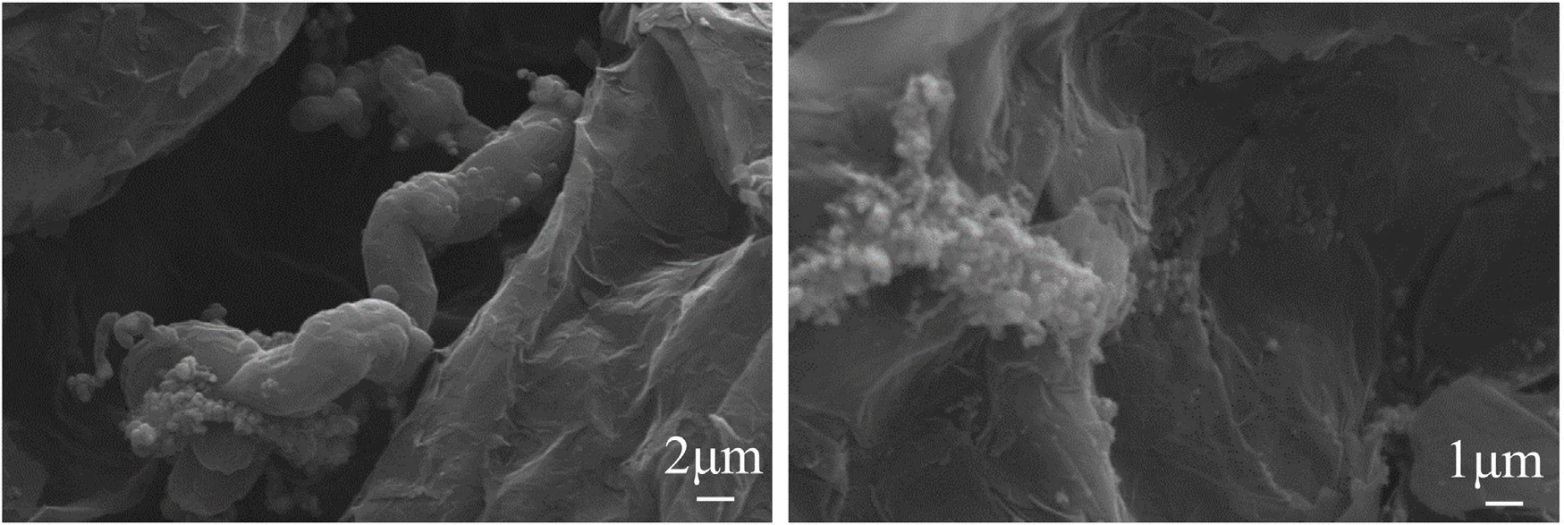
SEM and EDS images of sample 1.

The SEM images of sample 1 are shown in Figure 3. The, EG maintained a loose and vermicular morphology. Obviously, the CNT was not generated between the clearance in the, EG but granular material had been there, and the Co element and C element existed in Sample 1 according to the result of energy dispersive spectroscopy (EDS) analysis. It could be concluded that CNTs could not generate under the condition of the Co(NO_3_)_3_·6H_2_O catalyst without carbores P.

**FIGURE 4:**
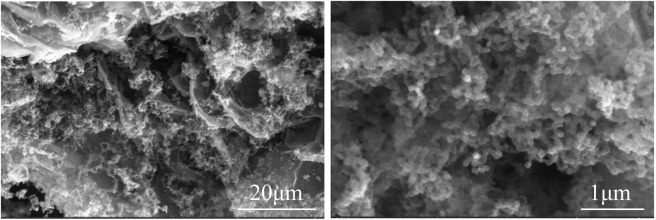
SEM images of sample 2.

The SEM images of sample 2 are shown in Figure 4. The irregular precipitate formed between the pores of expanded graphite. Without the Co(NO_3_)_3_·6H_2_O catalyst, the carbon nanotubes were not formed instead of the granules precipitate, which indicated the CNTs were not sufficiently grown in the sample without a Co(NO_3_)_3_·6H_2_O catalyst but with carbores P.

**FIGURE 5:**
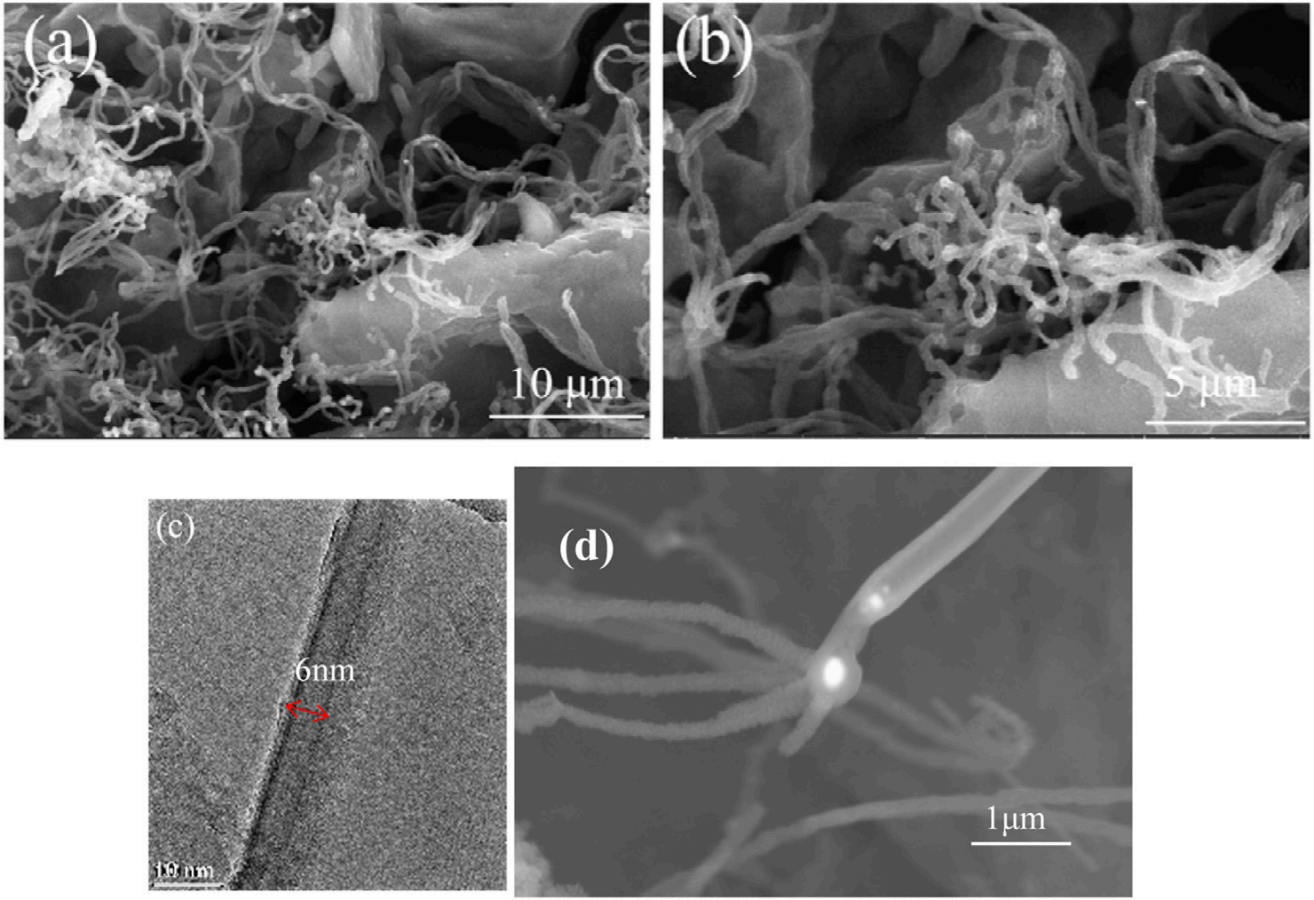
SEM **(A,B and D)** and TEM **(C)** images of sample 3.

The SEM and transmission electron microscopy (TEM) images of sample 3 are shown in Figure 5. There were a lot of curly fibers between the pores of expanded graphite, and the fibers were hollow and about 6 nm, which are carbon nanotubes (CNTs). In addition, the catalyst cobalt was located at the top of the carbon nanotubes, which lead to the formation of carbon nanotubes radially. As shown in Figure 5C, the nanotubes were typical of carbon nanotubes (CNTs) with a hollow structure.

### 3.3 Thermal decomposition of carbores P and expanded graphite

**FIGURE 6:**
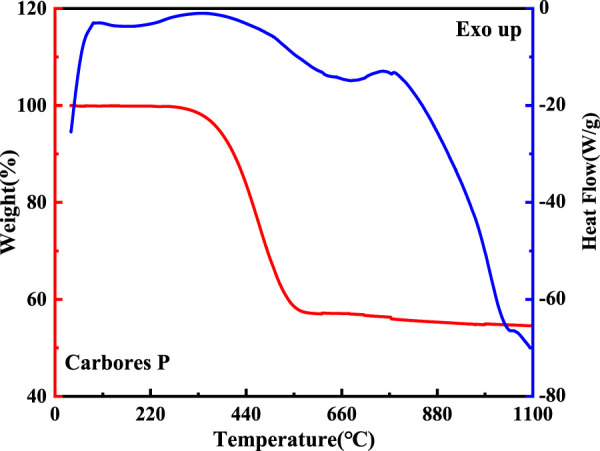
DTA-TG curve of carbores P.

**FIGURE 7:**
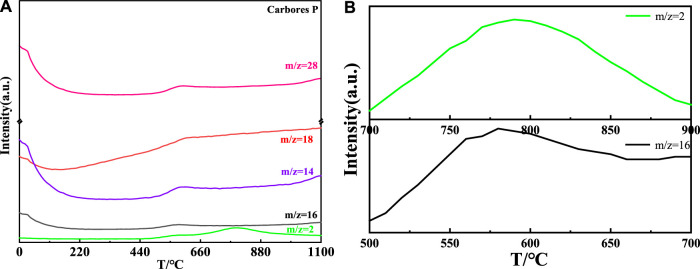
MS curve of carbores P **(A)** and local magnification of H_2_ and CH_4_ **(B)**.

**FIGURE 8:**
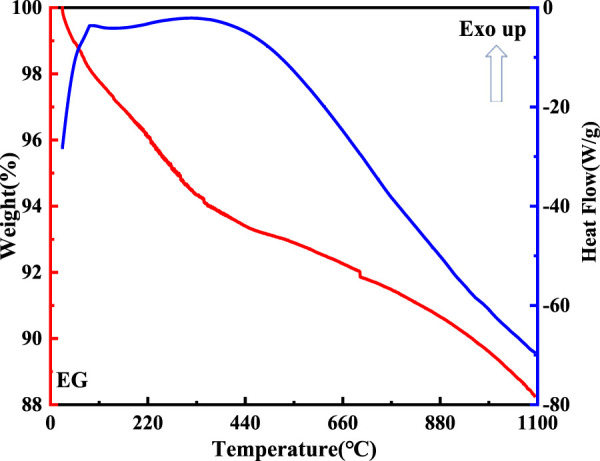
DTA-TG curve of the expanded graphite.

**FIGURE 9:**
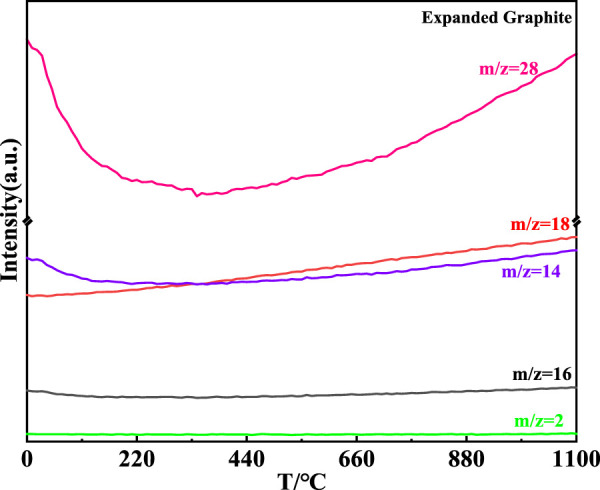
MS curve of the expanded graphite.

The thermal decomposition process of carbores P and expanded graphite was surveyed by DTA-TG-MS, and is shown in Figures 6–9.

The DTA-TG curve of carbores P is shown in Figure 6. Firstly, there were three endothermic peaks which were located at 168°C, 340°C, and 800°C in the DTA curve. This indicated that gas production started and the carbon began to resolve, as the TG curve showed a weight change from 97% to 57% between 340°C and 558°C. This phenomenon verified that the carbon source from the carbores P had decomposed to generate the gas.

The MS curve of carbores P is shown in Figure 7 (a). It could be concluded that the CH_4_, H_2_O, CO, and H_2_ gas corresponding to the m/z = 16, m/z = 18, m/z = 28, and m/z = 2, respectively, were generated at 558°C (Efimova et al., 2010; Hotová and Slovák, 2016), which was same as the TG-DTA curve. Interestingly, the local magnification of H_2_ showed that the H_2_ was produced at 700°C–900°C. Additionally, the MS curve of the CH_4_ gas presented distinct fluctuations in the local magnification. Similarly, in comparison with the DTA-TG curve, the MS curve was slightly undulating at 800°C. Through the comprehensive analysis of the MS and TG curves, it was found that carbores P began to produce CH_4_, H_2_O, CO, and H_2_ gas in this process.

The DTA-TG curve of the expanded graphite is shown in Figure 8. As for the DTA curve, reaction peaks formed at 100°C and 420°C. This indicated that volatile matter began to escape from the expanded graphite at 100°C. Additionally, it was remarkable that the weight of the expanded graphite changed from 100% to 88% in the TG curve due to its continuous decomposition.

The MS curve of the expanded graphite is shown in Figure 9. It could be concluded that the H_2_O and CO were generated with the continuous rise of temperature corresponding to the m/z = 18 and m/z = 28, respectively (Chien and Liao, 2020; Guo et al., 2022). Compared with the MS curve of carbores P, the intensity of the CO and CO_2_ generated in carbores P was more intense than that of the expanded graphite. Additionally, the production of the CH_4_ and H_2_ gases were not observed in, EG corresponding to the MS curve. The CH_4_ and H_2_ gases were actually involved in the reaction with the catalyst cobalt to form the growth of the CNTs, which demonstrated that the growth of the CNTs depended on the carbores P instead of the, EG. The results of the MS curve of the, EG was consistent with the SEM of the samples.

### 3.4 Valence analysis of Co

**FIGURE 10:**
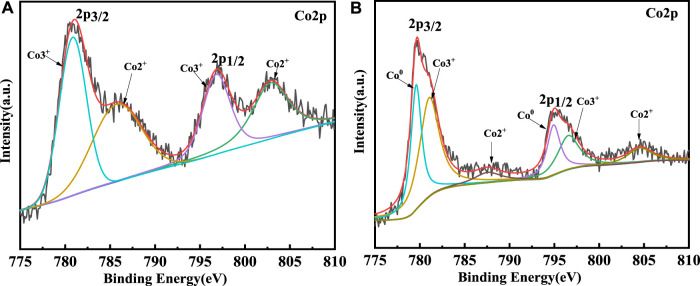
XPS surveys of sample 1 **(A)** and sample 3 **(B)**.

**FIGURE 11:**
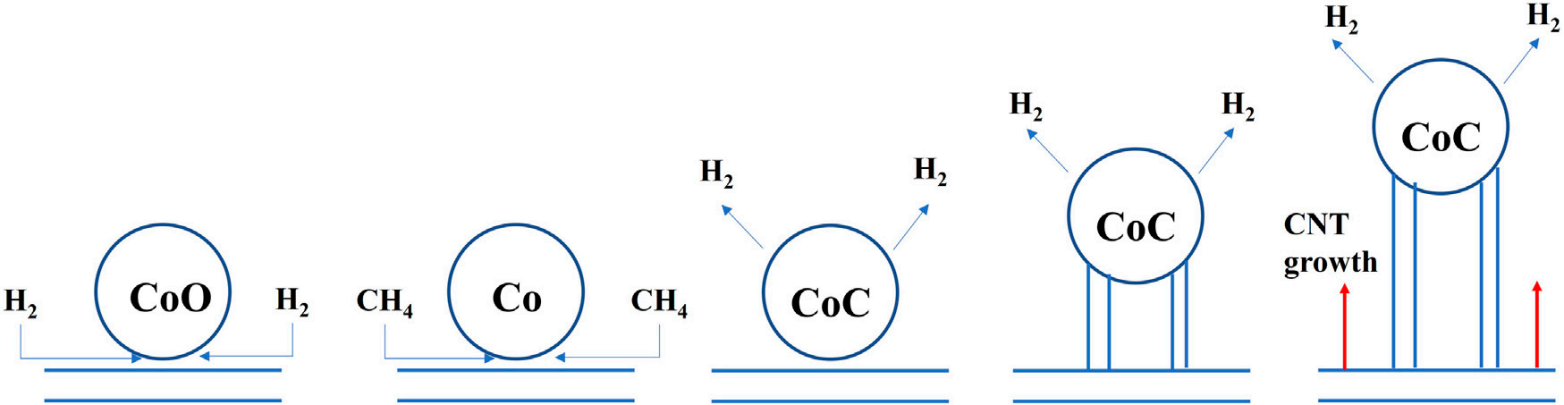
Schematic of the CNT growth mechanism.

The XPS was used to survey the valency of Co which would be changed in catalytic process. The XPS survey results of sample 1 and sample 3 are shown in Figure 10. Firstly, for sample 1, the curve could be fitted by two spin-orbit doublet characteristics of Co^3+^ and Co^2+^ in the XPS narrow survey of Co2p (Yang et al., 2019; Gao et al., 2021). Thus, there were four peaks corresponding to two valence states of Co, which were located at 780.6 eV and 796.8 eV and could be ascribed to Co^3+^, while the peaks located at 785.7 eV and 803 eV were attributed to Co^2+^. However, the Co 2p spectrum of sample 3 could be fitted in six peaks. The signals located at 779.9 eV and 794.8 eV could be attributed to 2p 3/2 and 2p 1/2 of Co^0^, respectively. The two peaks of 781.2 eV and 796.8 eV could be ascribed to the Co^3+^. The peaks at 787.9 eV and 804.7 eV could be assigned to Co^2+^. Through the contrastive XPS surveys of sample 1 and sample 3, it is worth mentioning that Co^0^ existed in sample 3. In general, in the process of CNT growth, Co^3+^ and Co^2+^ are reduced into metallic cobalt, which is reduced by the H_2_ gas. Thus, the Co^0^ could be detected in the sample 3. Additionally, Co2p_1/2_ was produced by CoO, which indicated the Co-O band existed (Liu et al., 2021). The results of the XPS coincided with the CNT growth mechanism. In theory, the growth process of CNTs could be revealed by Equations 1, (2). Firstly, the CoO is reduced by H_2_ to generate Co particles. Then, the Co particles react with the CH_4_ to generate CoC. At this time, the concentration of C atoms on the surface of the CoC is larger than the concentration of C atoms on the surface on the, EG. Thus, the C atoms should continuously spread due to the concentration difference of C atoms. The concentration difference of C atoms leads to the growth of CNTs. The schematic of the CNT growth mechanism is shown in Figure 11.

$$CoO+H_{2}\to Co+H_{2}O$$ (1)

$$Co+{CH}_{4}\to CoC+2H_{2}$$ (2)

### 3.5 Growth mechanism of CNTs

**FIGURE 12:**
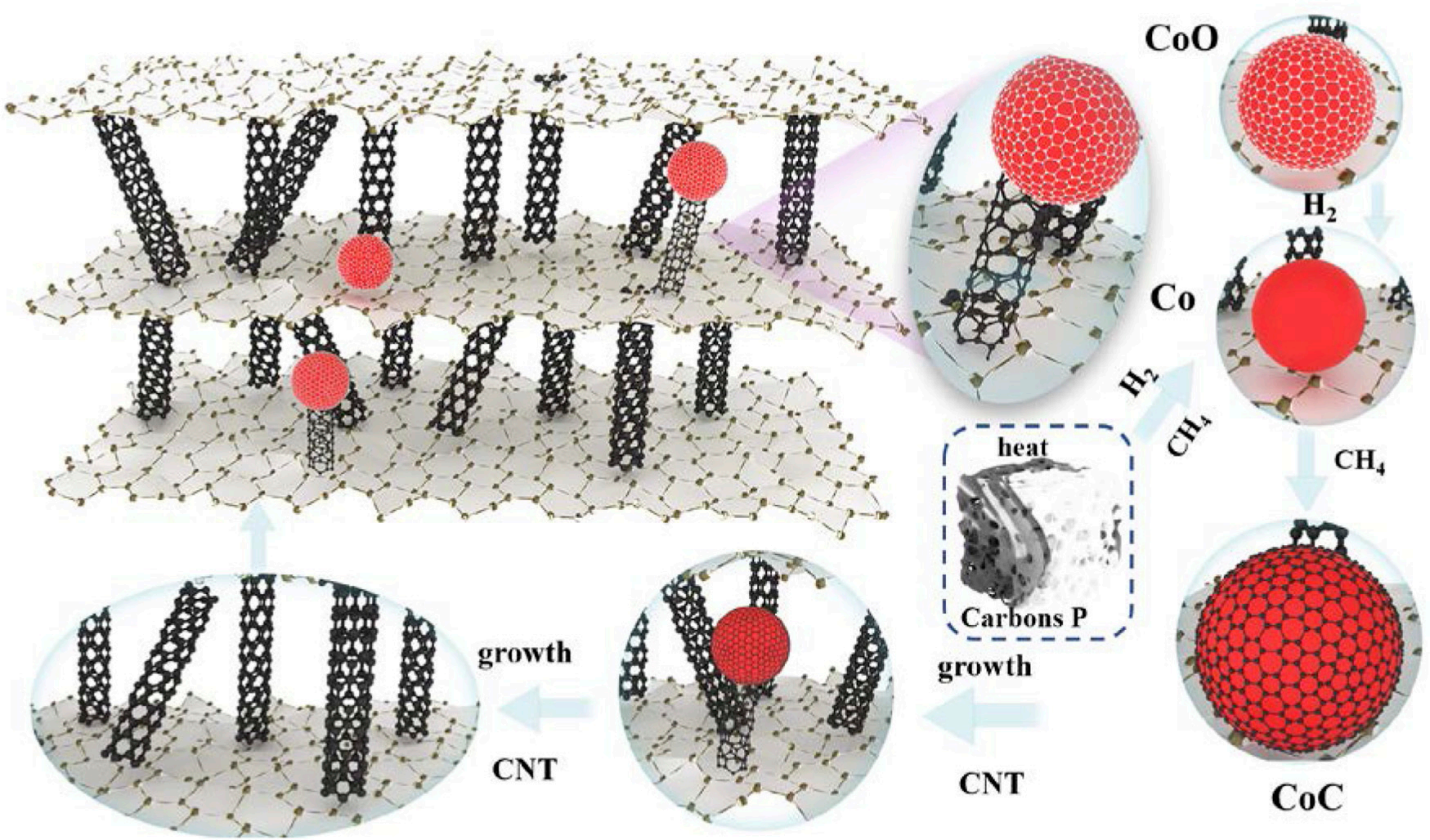
Schematic illustration of the CNT growth mechanism in, EG.

The schematic illustration of the CNT growth mechanism in, EG is shown in Figure 12. As is well known, the growth mechanism of carbon nanotubes for the synthesis of hydrocarbons from transition metal catalyzed thermal decomposition is not very clear, and the side emphasis of their accounts is different. This can be summarized as the “dissolution diffusion precipitation” model. Firstly, the carbon after the decomposition of hydrocarbons contacts with the bare metal surface. After that, dissolved carbon diffuses, disperses, and is transported to other sites of metal particles. Finally, carbon is deposited and precipitated at these site in the form of carbon nanotubes.

In this work, carbores P was used as the source of the C element. First of all, the expanded graphite was heated from room temperature to 1,100°C. In this process, many oxygenated functional groups were generated on the surface of the graphite crystal and, when the expanded graphite was heated to an elevated temperature, most of the oxygenated functional groups were decomposed into CO_2_ or H_2_O, leaving the high energy unsaturated carbon dangling bonds on the surface of expanded graphite. These unsaturated carbon dangling bonds became the active sites. At the same time, carbores P cleaved to produce hydrocarbon gases such as methane and hydrogen gas at an elevated temperature. Subsequently, the hydrogen gas reduced CoO to Co particles. Next, the CoO reacted with the CH_4_ to generate the CoC. The C atom contacted with the CoC and dissolved, adsorbed on the active sites of expanded graphite. Additionally, dissolved supersaturated carbon diffused in the CoC and was transported to other sites of the CoC. Ultimately, the concentration of C atoms on the surface of the CoC was larger than the concentration of C atoms on the surface on the, EG. The C atoms should continuously spread at these sites to generate carbon nanotubes. Then, the catalyst particles gradually became inactive and the carbon nanotubes stopped growing.

## 4 Conclusion

CNTs successfully grew in, EG with the synergistic effect of carbores P and the Co(NO_3_)_3_·6H_2_O catalyst. The growth mechanism of carbon nanotubes was that the heated carbores P produces hydrocarbon gas (methane and hydrogen gas), which reacted with the Co(NO_3_)_3_·6H_2_O catalyst to reduce CoO_x_ to Co particles, then the carbon form pyrolysis was deposited the on the surface catalyst Co particles, after continuous solid dissolution and precipitation, carbon nanotubes were at last generated in the, EG.

## Data Availability

The original contributions presented in the study are included in the article, further inquiries can be directed to the corresponding author.
